# ACAP-A/B Are ArfGAP Homologs in *Dictyostelium* Involved in Sporulation but Not in Chemotaxis

**DOI:** 10.1371/journal.pone.0008624

**Published:** 2010-01-07

**Authors:** Pei-Wen Chen, Paul A. Randazzo, Carole A. Parent

**Affiliations:** Laboratory of Cellular and Molecular Biology, National Cancer Institute, Bethesda, Maryland, United States of America; University of Birmingham, United Kingdom

## Abstract

Arfs and Arf GTPase-activating proteins (ArfGAPs) are regulators of membrane trafficking and actin dynamics in mammalian cells. In this study, we identified a primordial Arf, ArfA, and two ArfGAPs (ACAP-A/B) containing BAR, PH, ArfGAP and Ankyrin repeat domains in the eukaryote *Dictyostelium discoideum*. In vitro, ArfA has similar nucleotide binding properties as mammalian Arfs and, with GTP bound, is a substrate for ACAP-A and B. We also investigated the physiological functions of ACAP-A/B by characterizing cells lacking both ACAP-A and B. Although ACAP-A/B knockout cells showed no defects in cell growth, migration or chemotaxis, they exhibited abnormal actin protrusions and ∼50% reduction in spore yield. We conclude that while ACAP-A/B have a conserved biochemical mechanism and effect on actin organization, their role in migration is not conserved. The absence of an effect on *Dictyostelium* migration may be due to a specific requirement for ACAPs in mesenchymal migration, which is observed in epithelial cancer cells where most studies of mammalian ArfGAPs were performed.

## Introduction

Eukaryotic cells contain complex internal organelles enclosed within membranes and a cytoskeleton, which differentiate them from prokaryotic cells. These organelles provide compartmentalization for various metabolic activities. The cytoskeleton is a network of protein filaments extending throughout the cytoplasm to provide the structural framework of the cell. Remodeling of membranes and the actin cytoskeleton is essential for a variety of eukaryotic cell functions including protein secretion, maintenance of cell morphology and cell movement. Protein machineries that affect and regulate changes in membrane and actin structures have been identified and the coordination of the action of these proteins is being studied. Among them are the Arf family of GTP-binding proteins and the accessory proteins that regulate them, guanine nucleotide exchange factors (GEFs) and GTPase-activating proteins (GAPs) [1], [2].

The Arfs are a highly conserved family of GTP-binding proteins found in eukaryotes. The Arfs have a similar fold as other members of the Ras-superfamily of GTP-binding proteins but have the unique structural feature of an N-terminal extension that is covalently modified by myristic acid and can form an amphipathic helix when associated with membranes [3], [4], [5]. The Arf proteins in mammals are divided into three classes based on amino-acid sequence identity [6]. Class I Arfs (Arf1, Arf2 and Arf3) mainly localize to the Golgi apparatus although they also function in endosomal compartments. Class III Arf contains only Arf6, which is thought to function at the plasma membrane and a subset of endosomes [1]. The function of class II Arfs (Arf4 and Arf5) has not been extensively investigated. The primitive eukaryote *Giardia lamblia* has a single Arf. A second gene arose in fungi, which have one or two Arfs that are predecessors of metazoan class I/II Arfs and one Arf that is considered a class III Arf. Flies and worms have one Arf of each class. Humans have 5 Arfs: 2 class I (Arf1 and 3), 2 class II (Arf4 and 5) and 1 class III (Arf6). Other mammals have a third class I Arf (Arf2). The function of Arf proteins depends on their cycling between GTP- and GDP-bound forms. The exchange of GDP for GTP bound to Arfs is induced by ArfGEFs whereas hydrolysis of the bound GTP is mediated by ArfGAPs.

Thirty-one ArfGAPs have been identified in humans. They contain a common catalytic domain but are otherwise structurally diverse. Based on both domain structure and phylogenetic analysis of the ArfGAP domains, the family can be divided into 10 subfamilies [7]. Most ArfGAP subfamilies arose in metazoans, after the division of Arfs into three classes. Four subfamilies of ArfGAPs have been implicated in cell adhesion and migration, including ASAP-type and ACAP-type ArfGAPs [8]. The distinguishing structural features of ASAPs and ACAPs are a tandem of BAR (Bin, amphiphysin and Rvs161/167), PH (plekstrin homology), ArfGAP and Ank (Ankyrin repeats) domains. ASAPs also have an SH3 domain. In mammalian cells, ASAPs have been found to associate with focal adhesions, invadopodia, and circular dorsal ruffles where they regulate the structures and the associated actin cytoskeleton [9]. ACAPs also affect actin structures and ACAP1 regulates β1 integrin recycling [10], [11]. Although changes in ASAP and ACAP expression have been shown to affect cell migration, presumably through effects on actin and adhesion, the molecular mechanism by which they regulate cell migration has yet to be determined. In addition, some discrepancies have been reported in the literature. ASAP1 stimulates migration or invasion in some cases but has no effect in others [12], [13], [14], [15], [16], [17]. We therefore sought to more specifically study the role of ASAP/ACAPs in cell migration using the model organism, *Dictyostelium discoideum*. The intermediate complexity of *Dictyostelium* genome/proteome compared with yeasts and animals, together with its accessible genetics would likely allow us to gain more insights into the physiological functions of ArfGAPs and the molecular bases for their functions.

The social amoeba *Dictyostelium* is a useful model system for studying cell motility, phagocytosis, and vesicle trafficking [18]. *Dictyostelium* exists in two distinct life stages, a vegetative growth stage and a developmental stage. In their vegetative stage, *Dictyostelium* grow as single cells feeding on bacteria or defined media. When the food source is depleted, *Dictyostelium* cells enter a developmental program that allows them to form an aggregate by chemotaxing towards cAMP signals. The aggregate undergos dramatic morphological changes and differentiates into a multicellular structure including an optional slug that can migrate towards light and temperature gradients. After ∼24 hrs, the aggregate eventually forms a fruiting body composed of dormant spores atop a stalk of vacuolated cells [19], [20], [21]. The *Dictyostelium* genome has a single Arf gene, called *arfA*, that is similar to the primordial Arf in *Giardia* and 12 putative ArfGAP genes. Two ArfGAP genes are predicted to encode proteins containing BAR, PH and Ank domains in a similar configuration found in mammalian ASAPs and ACAPs. The role of ArfGAPs and Arfs in *Dictyostelium* has not been explored.

We set out to determine if the proteins structurally homologous to mammalian Arfs and ACAP/ASAPs have analogous biochemical and/or biological function in *Dictyostelium*. We isolated the *Dictyostelium arfA* and two ASAP/ACAP-type ArfGAP (*acap-A* and *B*) cDNAs. Recombinant ArfA, ACAP-A and ACAP-B were expressed in and purified from bacteria, and used in an in vitro system to assess their biochemical properties. To further understand the physiological functions of the ASAP/ACAP-type ArfGAPs, we disrupted *acap-A* and *B* genes in *Dictyostelium* by homologous recombination and generated *acap-A/B* single and double knockout cells. We found that *acap-A/B* double knockout cells exhibited abnormal actin protrusions and reduced spore yield but had no defects in chemotaxis.

## Results

### ACAP-A/B Are ArfGAP Homologs in Dictyostelium

**Figure 1:**
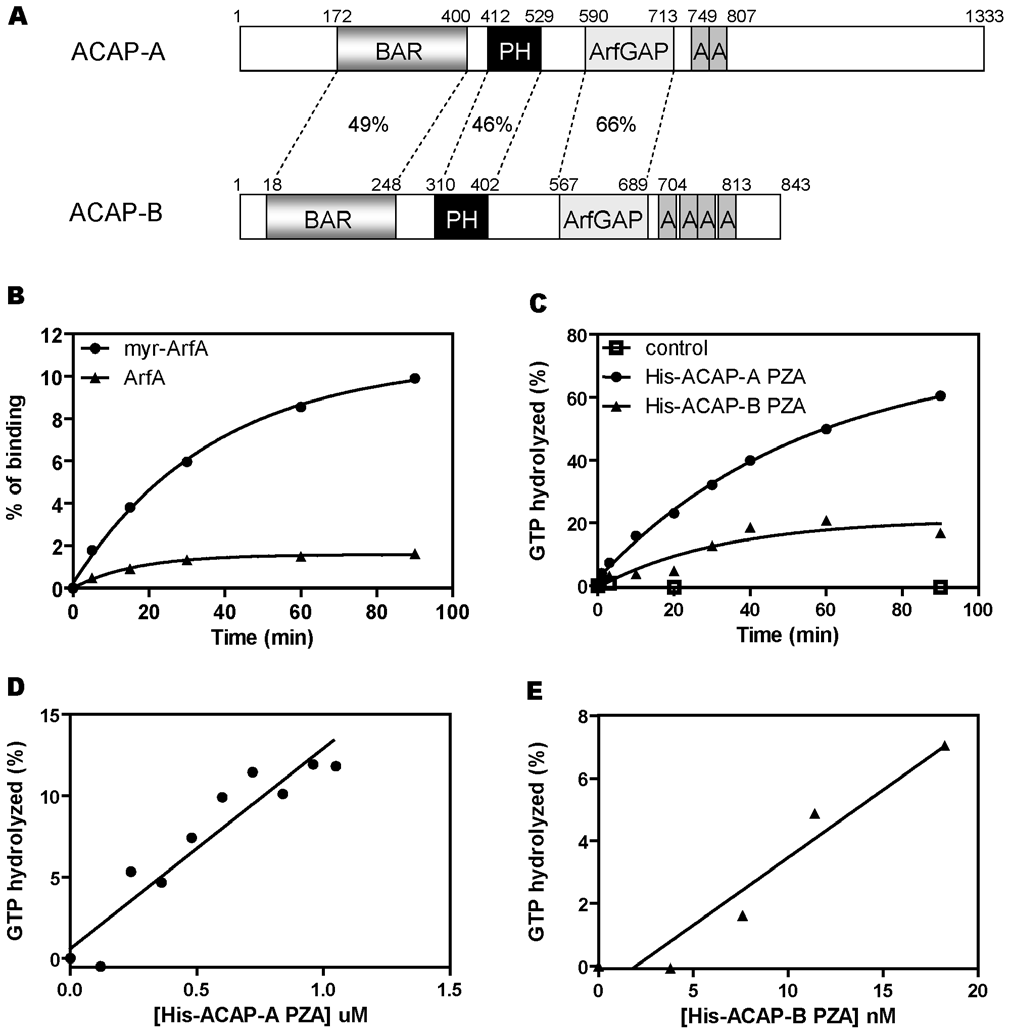
Identification of ACAP-A and ACAP-B as ArfGAP homologs in *Dictyostelium*. A, a schematic illustration of ACAP-A and ACAP-B domains. The structure of ACAP-A and ACAP-B was predicted by Pfam database of conserved protein domains. Amino acid sequence identity between each domain of the two proteins is indicated in percentages. B, GTPγS binding to myristoylated (myrArfA) and non-myristoylated ArfA was determined as described under “Methods.” 10 pmol of ArfA or myrArfA was used for each point. Data shown were the average of two experiments. C, time course of ArfGAP activity of *Dictyostelium* ACAP PZA proteins. His-ACAP-A PZA (0.9 µM) or His-ACAP-B PZA (22.8 nM) was incubated with 0.1 µM [α-^32^P]GTP-loaded myrArfA at 30°C. Aliquots were withdrawn at the indicated times and GTP hydrolysis measured as described under “Methods.” Data shown were the average of two experiments. D and E, dose dependence of *Dictyostelium* ACAPs. Different concentrations of His-ACAP-A PZA (D) or His-ACAP-B PZA (E) were incubated with 0.1 µM [α-^32^P]GTP-loaded myrArfA at 30°C for 90 min. Results shown were the average of at least two experiments.

To identify ArfGAPs in *Dictyostelium*, the ArfGAP domain of the human ASAP1 was used to search for homologous proteins in the *Dictyostelium* genome database. There are 12 genes encoding ArfGAP-containing proteins (Fig. S1). Based on protein domain predictions from the Pfam database, we identified two genes (DDB0233652 and DDB0233653) that encode proteins containing BAR, PH, ArfGAP and Ankyrin repeat domains. This domain structure resembles mammalian ASAPs and ACAPs (Fig. 1A). We compared the primary sequence of the ArfGAP domain of these two proteins with the reported yeast and mammalian ArfGAPs and found that they share the highest sequence homology to ACAPs (40–47% identity) and ASAPs (30–39% identity). Since these two proteins have a slightly higher homology to human ACAPs than ASAPs and do not contain a SH3 domain, they are called ACAP-A (DDB0233652) and ACAP-B (DDB0233653). ACAP-A contains a long (∼526 a.a.) carboxyl-terminal tail after the ankyrin repeats, which is characteristic of human ASAPs. In contrast, ACAP-B has a short carboxyl terminus similar to human ACAPs.

To determine if ACAP-A and ACAP-B have ArfGAP enzymatic activity, we measured the hydrolysis of GTP bound to Arf as described under “Methods.” Because mammalian ASAPs and ACAPs have different substrate specificity towards class I/II and class III Arfs, we first searched for Arf homologs in the *Dictyostelium* genome. ArfA, previously identified as a phagosomal protein [22], is highly homologous to class I and II human Arfs (83% identity) and contains the signature motifs that define Arf family proteins, including wDvGGqxxxRxxW and an extended N-terminus with a myristoylation site (Fig. S2). *ArfA* cDNA was isolated by RT-PCR from wild-type AX2 cells, expressed in and purified from bacteria and tested for its ability to bind GTP. For non-myristoylated ArfA, steady-state binding of GTPγS reached a stoichiometry of ∼1.6% of protein in the presence of LUVs (Fig. 1B triangles). N-myristoylation of ArfA (myrArfA) increased its GTPγS binding to ∼10% (Fig. 1B circles) [23], [24]. We conclude from these data that ArfA exhibits the biochemical properties similar to those of mammalian Arfs.

In mammalian ASAPs, the minimum structure that has detectable ArfGAP activity is comprised of the PH, ArfGAP and Ank repeat domains (abbreviated PZA for PH, zinc binding motif and Ank repeats) [25]. We therefore constructed bacterial expression plasmids encoding His-tagged PZA from ACAP-A (His_10_ [400–845]ACAP-A) and ACAP-B (His_6_ [307–843]ACAP-B). Proteins expressed in and purified from bacteria were tested for ArfGAP activity. Like human ACAPs, His-tagged PZA of ACAP-A and ACAP-B were insoluble when expressed in bacteria, regardless of the induction temperature. Therefore, His_10_ [400–845]ACAP-A or His_6_ [307–843]ACAP-B were purified from inclusion bodies in 6 M guanidine-HCl and refolded by dialysis. As shown in Fig. 1C–E, myrArfA alone had no detectable GTPase activity and His_10_ [400–845]ACAP-A and His_6_ [307–843]ACAP-B stimulated GTP hydrolysis by myrArfA in a time- and dose-dependent manner. These results demonstrate that ACAP-A and B have ArfGAP enzymatic activity.

### acap-A^−^/B^−^ Cells Do Not Exhibit Apparent Developmental Defects

**Figure 2:**
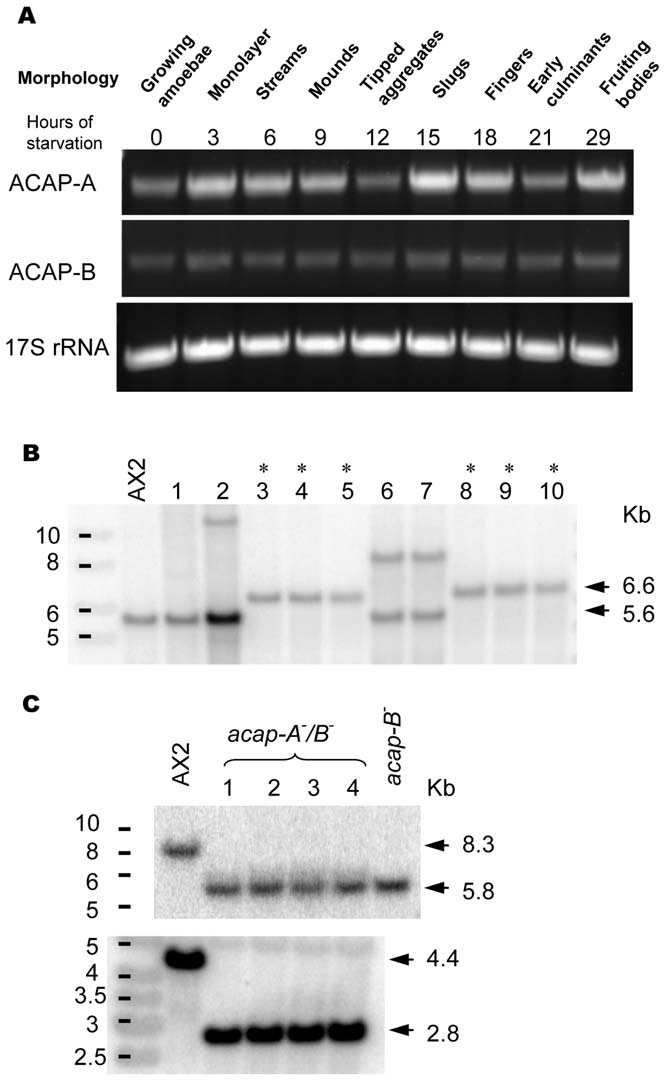
Generation of *acap-A^−^/B* ^−^ cells. A, Wild-type AX2 cells were allowed to develop on nutrient deficient agar. Total RNA was extracted using TRIzol Reagent at the times indicated. Specific cDNA fragments corresponding to *acap-A* gene (3121–4002 nt) and *acap-B* gene (1757–2532 nt) were amplified by RT-PCR and analyzed on agarose gels. 17S rRNA (1124–1714 nt) was analyzed in parallel as an internal control. B, Southern blot analysis of EcoRV digested genomic DNA from wild-type (AX2) and *acap-A* null cells (*acap-A^−^*) using the 5′ fragment of *acap-A* as the probe to confirm the targeted recombination (Fig. S3). * indicates clones with the targeted disruption of *acap-A* gene. *acap-A* null (clone 8) was subsequently used to generate *acap-A^−^/B* ^−^ cells. C, lower panel, Southern blot analysis of BglII/EcoRI digested genomic DNA from wild-type (AX2) and *acap-A^−^/B* ^−^ cells using the 5′ fragment of *acap-A* as the probe to confirm the loss of Bsr cassette upon transient Cre expression in *acap-A* null cells; upper panel, Southern blot analysis of BglII digested genomic DNA from (AX2), *acap-B^−^*and *acap-A^−^/B* ^−^ cells using the 5′ fragment of *acap-B* as the probe to confirm the targeted disruption of *acap-B* gene. 10 and 4 independent clones were analyzed in B and C respectively.

To understand the biological functions of ACAP-A and ACAP-B, we first examined the timing of their expression during the *Dictyostelium* life cycle by RT-PCR. We found that both ACAP-A and ACAP-B were expressed throughout development (Fig. 2A). While ACAP-B was expressed constantly at all developmental stages, ACAP-A expression was elevated during aggregation, in slugs and fruiting bodies. This result suggested that ACAP-A and ACAP-B could function at multiple developmental stages, especially during aggregation and fruiting body formation. Next, we generated single and double knockouts of *acap-A* and *acap-B* in order to determine their physiological functions. Since *acap-B* is located on chromosome 2, we chose the AX2 strain that lacks the chromosome 2 duplication as the parental cell line. Both *acap-A* and *acap-B* genes were disrupted by homologous recombination using the blasticydin-resistant cassette (Fig. S3). Due to the AT-rich and non-specific nature of *acap-B* gene 5′ nucleotide sequence, we were unable to obtain a complete *acap-B* null strain but generated a mutant, *acap-B* ^−^, that retains the BAR domain (residues 1–290). Nevertheless, *acap-B* ^−^ deletes more than 65% of ACAP-B including the catalytic core (PZA domain) and therefore should not have ArfGAP activity or bind Arf. ACAP-A and ACAP-B share significant sequence identity in multiple domains (Fig. 1A), suggesting that they might have redundant functions. Therefore, double knockout cells, *acap-A^−^/B* ^−^, were generated by disrupting *acap-B* in an *acap-A* null background (*acap-A^−^*) using the cre/loxP method described previously (Fig. S3) [26]. The disruption of the genes was confirmed by PCR (data not shown) and Southern blot analysis on the genomic DNA of individual clones (Fig. 2B and C).

Preliminary characterization of *acap-A^−^*, *acap-B* ^−^and *acap-A^−^/B* ^−^ cells revealed no apparent abnormality. Single or double knockout cells grew normally in suspension and on bacterial lawns. Knockout vegetative cells showed normal cell morphology and the size of the bacterial lawn plaques from knockout cells was not different from wild-type (data not shown). Together, these data suggest that ACAP-A and ACAP-B are not crucial for cell growth or phagocytosis.

**Figure 3:**
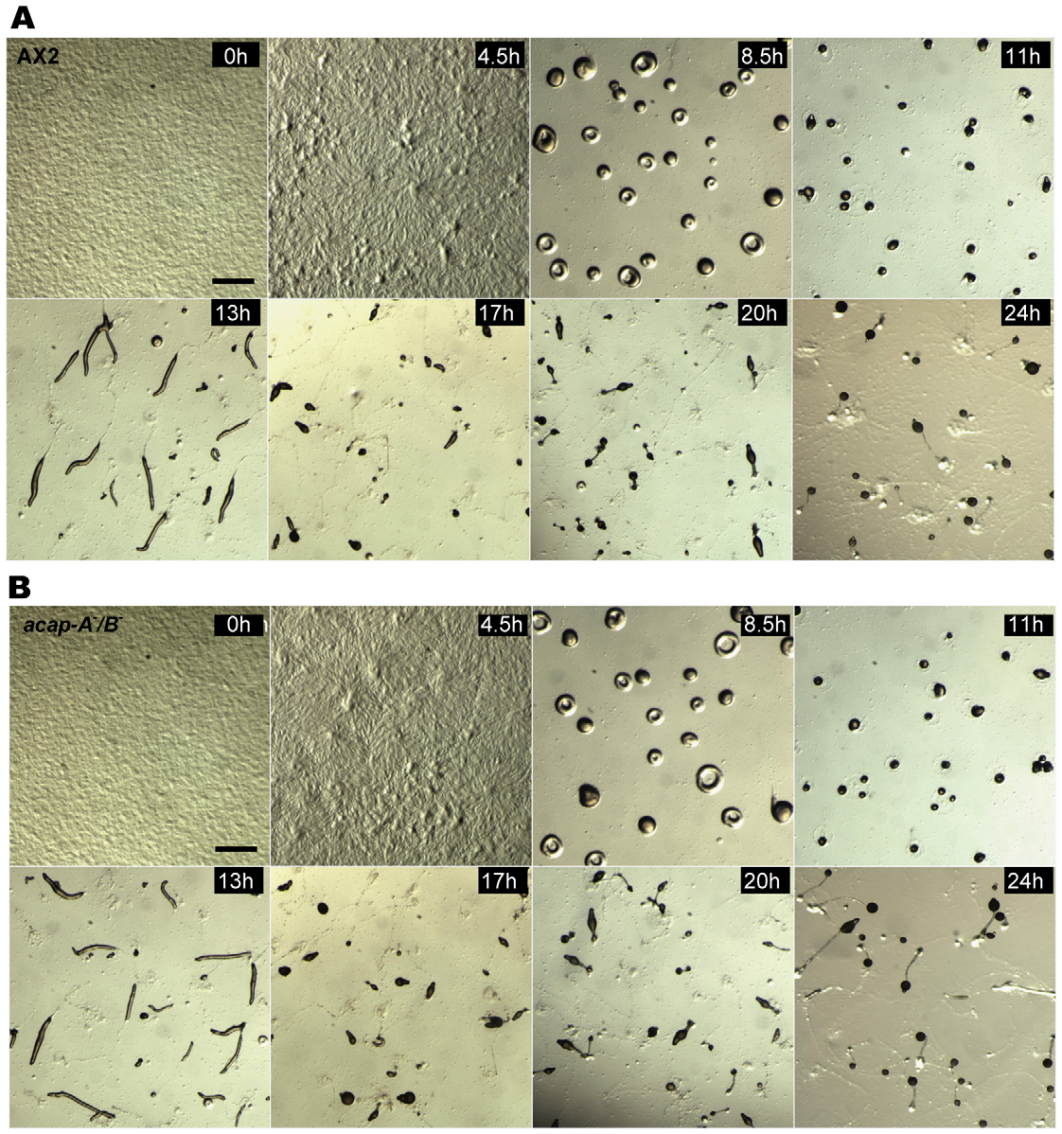
ACAP-A/B do not regulate *Dictyostelium* early development and fruiting body formation. Wild-type AX2 (A) and *acap-A^−^/B* ^−^ cells (B) were allowed to develop on nutrient deficient agar. Images were taken at the times indicated after plating on agar using a Leica stereoscope. Representative images were shown. Bars, 1 mm.

Multicellular development involves many aspects of cellular functions such as adhesion, contractility and apoptosis. ArfGAP homologs in plants have been shown to regulate different stages of development [27], [28]. To investigate the involvement of ACAP-A and ACAP-B in multicellular development, we compared the ability of wild-type, *acap-A^−^*, *acap-B* ^−^ and *acap-A^−^/B* ^−^ cells to undergo development and form fruiting bodies on nutrient deficient phosphate agar. Single or double knockout cells entered the developmental program in a timely fashion, starting aggregation ∼5 h after the initiation of starvation. By ∼8.5 h, the hemispherical mounds formed by the aggregated cells were evident and continued to differentiate into tipped aggregates (11 h), slugs (13 h), early culminants (20 h) and ultimately fruiting bodies (24 h). The time course of the single or double knockout cells development was indistinguishable from wild-type cells (Fig. 3A and B, single knockouts not shown). Under the conditions used, there was no apparent difference in the number, size or shape of the fruiting bodies between wild-type and knockout cells (Fig. 3A and B, single knockouts not shown). We also compared the development of wild-type and *acap-A^−^/B* ^−^ cells on soil plates which more closely resemble the native environment. We found no apparent abnormality in the morphology of tip aggregates, the early culminants or fruiting bodies from *acap-A^−^/B* ^−^ cells on soil plates. The timing of the development was also similar to wild-type (data not shown). We conclude that *Dictyostelium* do not require ACAPs to initiate the developmental program and to form fruiting bodies.

### ACAP-A/B Are Involved in Spore Generation

**Figure 4:**
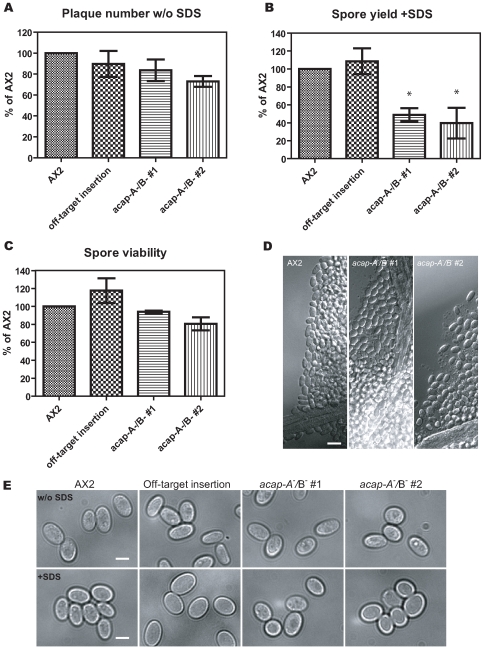
ACAP-A/B are involved in spore generation but not germination. 48 h after development on nutrient deficient agar, spores from wild-type, two independent clones of *acap-A^−^/B* ^−^ and one off-target insertion clone (Fig. 2B, clone 7) were collected and treated without or with 0.5% SDS as described under “Methods.” A and B, a fixed volume of spore suspension was plated on bacterial lawns. The number of germinating AX2 spores was 269±95 per µl after treated with SDS (B), which was about 89% of the germinating spores without SDS treatment (A). C, spore concentrations were counted and 30 spores were plated on bacterial lawns. The number of plaques formed from each sample was scored and expressed as a percentage relative to plaques formed by wild-type spores. Data shown were the mean±s.e.m. of at least three independent experiments. * indicates significantly different from wild-type by one way ANOVA using Dunnett's multiple comparison test, *p*<0.05. D, AX2 and *acap-A^−^/B* ^−^ fruiting bodies were isolated and spores in the sori were imaged by DIC microscopy. E, individual spores from AX2, off-target insertion and *acap-A^−^/B* ^−^ cells were harvested, treated as in (A) and (B), and viewed by DIC microscopy. Bars, 10 µm (D); 4 µm (E).

To complete a successful *Dictyostelium* life cycle, spores in the sori must be able to survive through harsh environmental conditions and germinate into single cell amoebae when suitable growth conditions are present [19]. To test if ACAP-A and B are involved in this stage of *Dictyostelium* development, we determined the spore yield of the wild-type and knockout cells. Since we did not observe any phenotype in single or double knockouts, we decided to focus on the double knockout cells for these experiments. The fruiting bodies on the agar were collected and an equal volume of the solution containing spores was plated on bacterial lawns. 3 days later, plaques on bacterial lawns were counted as a measure of the spore yield. Without detergent treatment, wild-type cells and *acap-A^−^/B* ^−^ cells generated comparable amount of spores (Fig. 4A). In contrast, when the spores were treated with detergent to mimic harsh conditions, we found that the spore yield of *acap-A^−^/B* ^−^cells was ∼50% of the wild-type cells (Fig. 4B). Two individual clones of *acap-A^−^/B* ^−^ cells had ∼50% reduced spore yield and an off-target gene clone had a similar amount of spores as wild-type, indicating that the reduced spore yield was not due to the selection process but a result of loss of ACAP-A and ACAP-B. A reduced spore yield may result from fewer cells differentiated into SDS-resistant spores, and/or the spores being less viable and unable to germinate. To test the possibility that ACAP-A and ACAP-B may play a role in germination, we plated a defined number of SDS-treated, morphologically intact spores on bacterial lawns and determined the spore viability. We found that wild-type and *acap-A^−^/B* ^−^spores grew similarly on bacteria, so as long as the *acap-A^−^/B* ^−^ spores remained intact after the detergent treatment, they can germinate and grow on bacterial lawn (Fig. 4C). Because the spore yield of *acap-A^−^/B* ^−^ cells was reduced only after SDS treatment, we reasoned that *acap-A^−^/B* ^−^ spores may have more fragile spore coats. Some *Dictyostelium* mutants with defective spore coats produce round spores [29], [30]. We therefore examined the morphology of the wild-type and *acap-A^−^/B* ^−^ spores before and after SDS treatment. As shown in Fig. 4D and E, the shape of the individual spores and spores in the spore heads was similar in wild-type and *acap-A^−^/B* ^−^ cells with or without SDS treatment. We conclude that ACAP-A and ACAP-B are important for spore generation and maturation but not germination. Taken together, our findings establish that ACAP-A and ACAP-B are specifically important for the spore formation in the late stage of *Dictyostelium* life cycle.

### ACAP-A/B Affect Actin Cytoskeleton

**Figure 5:**
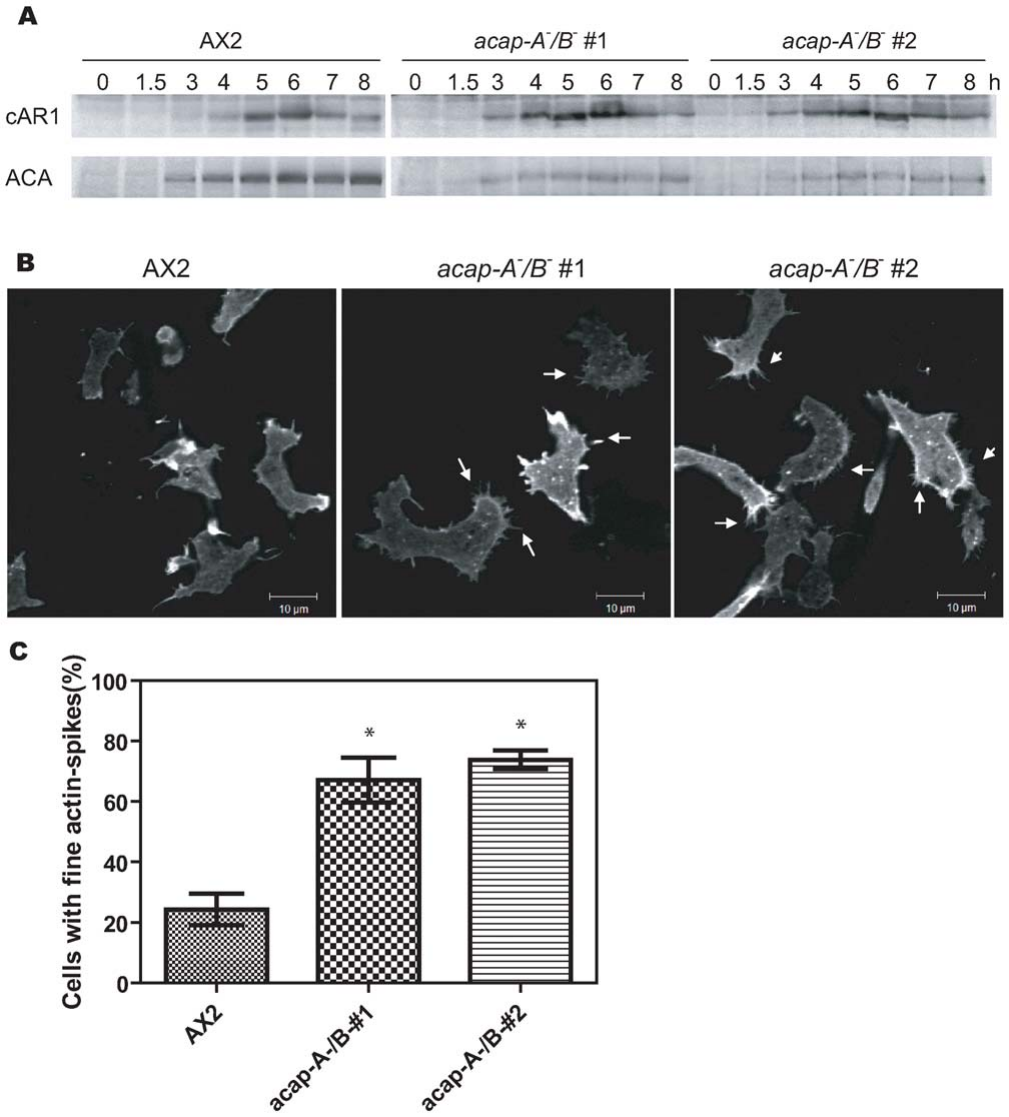
ACAP-A/B affect actin cytoskeleton. A, Western blot analysis of cAR1 and ACA expression after the onset of starvation with exogenous cAMP pulsing. Lysates from cells ministered with cAMP pulsing for various times were analyzed by immunoblotting using anti-cAR1 or ACA polyclonal antibodies. Representative blots from two experiments were shown. B and C, wild-type and two independent clones of *acap-A^−^/B* ^−^ cells were developed for 5–6 h, fixed and stained with TRITC-phalloidin to visualize F-actin. Results shown were representative images (B) and the quantification of the percentage of cells exhibiting fine actin protrusions (C, mean±s.e.m.) of three independent experiments. Cells were scored as “with fine actin spikes” when there were more than 6 actin protrusions in a cell. Typically, these cells exhibited more than 10 actin protrusions which were uniformly distributed around the periphery. In each experiment, a total of 60∼110 cells were analyzed for each cell line. Arrows indicate some actin-containing protrusions. * indicates significantly different from wild-type by one way ANOVA using Dunnett's multiple comparison test, *p*<0.05.

As several ArfGAPs including ACAPs and ASAPs have been shown to regulate the actin cytoskeleton [9], [10], [31], we set out to determine if ACAP-A and B affect the actin structures at different developmental stages using phalloidin staining of F-actin. We did not observe any difference in F-actin pattern between wild-type and *acap-A^−^/B* ^−^ vegetative cells (data not shown). We next studied F-actin organization in highly motile chemotaxis-competent cells. To obtain homogeneous cell populations for these experiments, cells were pulsed with exogenous cAMP every 6 min [32], [33]. Consistent with the result of normal development on non-nutrient agar, exogenous cAMP pulsing induced timely expression of the early developmental markers, cAR1 and ACA in *acap-A^−^/B* ^−^ cells as in wild-type (Fig. 5A). Interestingly, after 5–6 h of cAMP pulsing, *acap-A^−^/B* ^−^ cells demonstrated more fine protrusions compared with wild-type cells (Fig. 5B and C). These protrusions were usually shorter than those in wild-type cells and were present all around the cells. The difference in morphology of the actin protrusions was most prominent at the cell-substratum junction. Quantification of the 3D reconstruction images from F-actin staining revealed no difference in total F-actin amount between *acap-A^−^/B* ^−^ and wild-type cells. These data demonstrate that the regulation of the actin cytoskeleton is a conserved function of ACAPs in *Dictyostelium*.

### ACAP-A/B Do Not Affect Chemotaxis and Streaming

**Figure 6:**
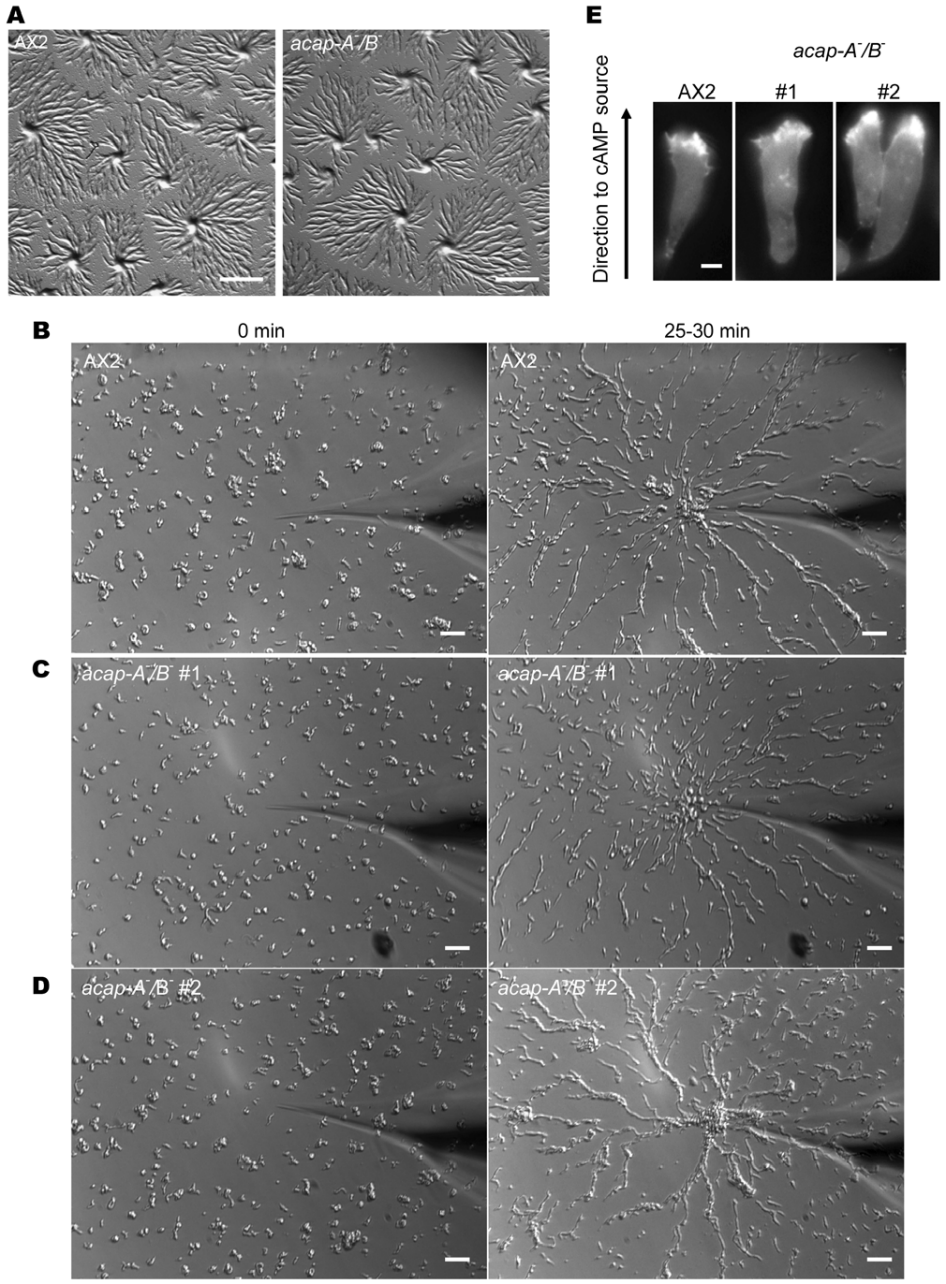
ACAP-A/B are dispensable for chemotaxis and streaming in *Dictyostelium*. A, images were taken 6 h after cell were plated on non-nutrient agar as described in Fig. 3. B–D, *acap-A^−^/B* ^−^ cells migrated towards exogenous cAMP and formed streams. Wild-type (B) or *acap-A^−^/B* ^−^ cells (C, D) were differentiated with cAMP pulses for 5–6 h and subjected to a point source of 1 µM cAMP. Images were taken every 10 sec by time lapse microscopy as described in “Methods.” Data shown were representative of at least five experiments with two independent clones of *acap-A^−^/B* ^−^ cells. Also see Videos S1–S4. E, representative images of wild-type AX2 or *acap-A^−^/B* ^−^ cells expressing mRFPmars-LimEΔcoil during chemotaxis in a gradient of cAMP. Bars, 1 mm (A); 25 µm (B–D); 6 µm (E).

Several mammalian ACAP/ASAP members have been implicated in stimulating cell migration and invasion, but the detailed mechanism responsible for their action is unclear and likely to be complex [11], [12], [13], [17]. We set out to test if ACAPs regulate cell migration in *Dictyostelium* and if they do, to understand their roles at a molecular level. Cell migration requires, among other things, remodeling of the actin cytoskeleton. Since we observed abnormal actin structures in *acap-A^−^/B* ^−^ cells (Fig. 5B and C), we wondered if these defects would also lead to alterations in migration. We first examined the ability of these cells to form aggregates on non-nutrient agar. Wild-type cells can relay chemotactic signals by secreting cAMP to neighboring cells. As a result, they align in a head-to-tail fashion to form long streams. Therefore, streaming can be used as a measurement for the ability to perform signal relay. Fig. 6A shows pictures taken 6 h after cells were plated on non-nutrient agar. As can be seen, *acap-A^−^/B* ^−^ cells were able to spontaneously migrate and aggregate similarly to wild-type cells, showing characteristic star-shape cell streams. This was apparent at both high (520000 cells/cm^2^, Fig. 6A) and low (52000 cells/cm^2^, data not shown) plating densities. These data show that ACAPs are not essential for *Dictyostelium* cell migration and chemotaxis to aggregation centers. In addition, as *acap-A^−^/B* ^−^ cells showed normal stream formation to aggregation centers upon starvation, these findings also demonstrate that ACAPs are not required for cells to generate and secret cAMP during signal relay. However, this assay does not allow the visualization of the morphological changes in individual migrating cells. To study the role of ACAPs in *Dictyostelium* motility, chemotaxis and streaming more carefully, we performed micropipette chemotaxis assays. In this assay, cells were pulsed with cAMP for 5–6 h and subjected to a cAMP gradient generated by a micropipette. Here again we found that *acap-A^−^/B* ^−^ cells migrated towards a point source of cAMP with no apparent change in speed or directionality compared with wild-type cells (Fig. 6B–D and Videos S1, S2, S3, and S4). In addition, *acap-A^−^/B* ^−^ cells exhibited a normal elongated shape when responding to a gradient of cAMP. Consistent with this polarized shape, *acap-A^−^/B* ^−^ cells showed normal F-actin accumulation in the leading edge as monitored by the localized distribution of the F-actin binding protein, mRFPmars-tagged LimEΔcoil [34], [35] (Fig. 6E). We also compared the ability of *acap-A^−^/B* ^−^ cells to form streams when chemotaxing to a point source of cAMP with wild-type cells and found that *acap-A^−^/B* ^−^ cells exhibited normal streaming behavior. The timing of stream formation, and the length and morphology of the streams were indistinguishable from those observed in wild-type cells although there are some clonal variations (compare Fig. 6C and D). We conclude that ACAP-A and ACAP-B are not essential for establishing polarity and migration during chemotaxis. Our data also establish that ACAP-A/B do not regulate signal relay during collective migration in *Dictyostelium*.

## Discussion

In this study, we identified two ArfGAP homologs, ACAP-A and ACAP-B, and examined their physiological roles in the eukaryotic organism *Dictyostelium*. ACAP-A and ACAP-B are multi-domain proteins with a BAR domain at the N terminus, followed by PH, ArfGAP and Ankyrin repeat domains. Like the mammalian ACAP/ASAP homologs, the truncated ACAP-A/B consisting of PH, ArfGAP and Ankyrin repeats can function as ArfGAPs in vitro using GTP bound myrArfA as a substrate. Functional analysis of the double knockout cells, *acap-A^−^/B* ^−^, revealed their roles in organizing the actin cytoskeleton and spore production. We did not find evidence for a role of ACAP-A or ACAP-B during chemotaxis and streaming.

Arfs are highly conserved throughout eukaryotes, maintaining >60% primary sequence identity [6]. *Giardia*, an organism thought to be a representative of the early diverging eukaryote lineage, has a primordial Arf that is ∼70% and 63% identical to human class I/II and class III Arfs [36]. Our sequence analysis identified ArfA as the only Arf in the *Dictyostelium* genome, with ∼83% and 66% identity to human class I/II and class III Arfs. Although ArfA was previously identified in a screen for phagosomal proteins [22], the biochemical properties of ArfA have not been examined. We found that *Dictyostelium* ArfA binds to GTP and it has no detectable GTPase activity. Myristoylation of ArfA increases its GTP binding stoichiometry. Whether or not ArfA has conserved in vivo function as a regulator of membrane trafficking in *Dictyostelium* remains to be determined. In the *Dictyostelium* genome, there are 3 proteins other than ArfA with significant (close to 60%) sequence identity with human Arf1 but they all lack a N-myristoylation site and have an N-terminus that is 8 amino acids longer than the N-terminus of Arf1 (Fig. S2). Characterization of these proteins in vitro and in vivo will determine if they can also function as Arfs.

Given the lack of intrinsic GTPase activity of ArfA, its activity is predicted to be regulated by GAPs. In this study, we identified two ArfGAPs, ACAP-A and ACAP-B, that can stimulate GTP hydrolysis by myrArfA. Compared with human ArfGAPs, ACAP-A/B appeared to have less enzymatic power. This could be due to the presence of partially unfolded proteins after the process of denaturing and refolding used to purify the protein. The low activity could also be the result of the assay conditions. *Dictyostelium* cells are usually grown at 22°C and will not grow above 28°C [37]. Therefore, assay conditions, including temperature, established for mammalian proteins may not be appropriate for the comparison of the human and *Dictyostelium* ArfGAPs.

The mechanisms by which *Dictyostelium* ACAPs regulate sporulation remain to be determined. A lower spore yield could be a result of fewer cells aggregating and therefore fewer cells in a mound to differentiate into spores. We found that the lack of ACAP-A/B did not give rise to defects in self-aggregation upon starvation or in chemotaxis towards exogenous cAMP. Therefore, the lower spore yield we measured in the ACAP-A/B double knockout cells is more likely due to defects in the post-aggregation processes. Moreover, the spore yield of the ACAP-A/B double knockout cells is reduced only after detergent treatment, strongly suggesting that these double knockout cells are normal in cell fate determination of prestalk and prespore cells but defective in spore coat formation. Indeed, several actin-binding protein mutants have been shown to have lower spore yield [29], [38] and we found *acap-A^−^/B* ^−^ cells exhibited abnormal actin protrusions. It is therefore possible that ACAP-A/B might regulate sporulation by organizing actin structures. Identification of the structural elements in ACAP-A/B responsible for the actin structural change and sporulation defect will test this hypothesis. Spore coat proteins are stored in and secreted from the prespore vesicles (PSVs). The mechanism of PSV formation and trafficking is largely unclear [39]. Proteomic analysis provided strong evidence for actin-based movement of the PSVs since actin and other actin-binding proteins are found in the purified PSVs [40]. Some *Dictyostelium* mutants known to have disorganized spore coats exhibit a round-spore phenotype [29], [30], but this abnormal spore morphology is not caused by defective PSV formation or secretion [29]. Therefore, although we did not observe any apparent spore shape change in *acap-A^−^/B* ^−^ cells, we can not exclude the possible role of ACAP-A and B in spore coat formation by regulating PSV formation and trafficking. Some human ACAP/ASAPs have been shown to regulate endocytic recycling [11], [41], [42] and an ACAP homolog in *Arabidopsis* functions in leaf vein patterning by regulating vesicle transport [27]. Therefore, it is possible that ACAP-A/B contribute to proper spore coat formation by mediating PSVs formation and/or trafficking dependent on the actin cytoskeleton.

Although mammalian ACAP/ASAP members including ACAP1, ASAP1 and ASAP3 have been shown to regulate cell migration [11], [12], [13], [17], the molecular bases for their function have not been described and several discrepancies in the literature have not been resolved. For example, reduction of ASAP1 expression has been shown to have no effect on cell migration and invasion in one study whereas reduced ASAP1 expression has been found to reduce cell migration and invasion in other studies [13], [14], [16], [17]. The discrepancies could be due to different assay conditions (2D versus 3D) or different cell types used. Therefore, we sought to test whether this type of ArfGAP can regulate motility in *Dictyostelium* and found that ACAP-A/B are not required for migration and chemotaxis. It has been recognized that there are different types of cell movements characterized by distinct molecular requirements [43], [44]. The role of ACAP/ASAPs in migration has only been examined in epithelial cancer cells or fibroblasts in which integrin-dependent movement is typically used [12], [13], [14], [15], [16], [17]. Mammalian ACAP/ASAPs are known to associate or regulate structures containing integrins and cadherins [9]. *Dictyostelium* does not contain strict homologs of these proteins. Therefore, regulation of motility by ACAP/ASAPs might be coupled to specific molecules but dispensable for integrin-independent movement. Further examination of ACAP/ASAPs function in other types of cells, such as leukocytes and T lymphocytes, whose movement can be independent of integrins [45], [46], or in epithelial cells with reduced integrin expression will test this hypothesis.

The role of ArfGAPs in the streaming behavior of *Dictyostelium* needs to be further studied. The ability of cells to form long streams and to perform chemotaxis can be dissociated. For example, adenylyl cyclase (ACA) null cells can migrate along a cAMP gradient but fail to form streams [47]. GxcDD is another putative *Dictyostelium* ArfGAP that contains calponin homology (CH), IQ motifs, RacGEF and PH domains in addition to an ArfGAP domain (Fig. S1) [48]. The ArfGAP domain of GxcDD contains a lysine in place of the catalytic arginine, strongly suggesting that GxcDD may not have ArfGAP activity. However, it may still participate in Arf signaling by functioning as an Arf effector since it might still bind Arf•GTP [25]. Our results show that ablation of two ArfGAPs, ACAP-A/B, had no effect on streaming. In contrast, GxcDD null cells had defects in streaming [48]. It is possible that domains other than ArfGAP present in GxcDD but not in ACAP-A/B might be crucial for streaming. GxcDD can bind to several Rac proteins, so the integration of Rac and Arf signaling may be essential for the streaming behavior in *Dictyostelium*.

Based on the results presented in this study, we conclude that ACAPs have conserved biochemical activity as an ArfGAP and affect actin organization in *Dictyostelium* with a possible physiologic function in the regulation of sporulation. We also conclude that ACAPs are not involved in the migration of *Dictyostelium* during development.

## Methods

### Cell Culture and Development

*Dictyostelium discoideum* wild-type strain AX2 and *acap-A^−^/B* (1–290) cells were cultured in HL-5 media at 22°C in Petri dishes or shaking flasks. *Acap-A^−^/B* (1–290) cells were supplemented with 10 µg/ml Blasticidin S (MP Biomedicals, Solon, OH). Cells were allowed to differentiate to the chemotaxis competent stage as described previously [32], [33]. Briefly, exponentially grown cells in suspension culture were harvested, washed and resuspended at 2×10^7^ cells/ml in development buffer (DB: 5 mM Na_2_HPO_4_, 5 mM KH_2_PO_4_, pH 6.2, 2 mM MgSO_4_, and 0.2 mM CaCl_2_). Cells were then given pulses of 75 nM cAMP every 6 min for 5–6 h. To verify that cells were developed to the chemotaxis competent stage, Western blot analysis was performed using antibodies against the early developmental markers, cAMP receptor 1 (cAR1) and the adenylyl cyclase (ACA) expressed during aggregation.

### Development on Agar

5×10^6^ cells were washed once, resuspended in DB and plated on DB agar in 6-well plates (DB with 1.5% agar). Cells were allowed to adhere for 15 min then the liquid was removed and the plates were incubated at 22°C for the times indicated. Cells were visualized at different time points on a Leica stereoscope.

### RNA, Genomic DNA Isolation, RT-PCR and Molecular Cloning

Total RNA was extracted from AX2 cells at different developmental stages using TRIzol Reagent according to the manufacturer's instructions. RT-PCR for determining the temporal expression pattern of ACAP-A and B was performed in two steps: first-strand cDNA was synthesized by SuperScript III Reverse Transcriptase followed by a PCR reaction with *Taq* DNA polymerase (Invitrogen, Carlsbad, CA). Primers used to amplify specific cDNA fragments of *acap-A* and *acap-B* genes were: 5′-GAGGGTGGTAGTGAATCATTAGCTCCAC-3′ and 5′-CCATTCTGATGCCAATACAAATGAATAA-3′ (*acap-A*); 5′-GTAACACGCGTGATCCAGATTGGGCATC-3′ and 5′-GTTAAGAGGTTTCGTTATTAATTGCAAATAA-3′ (*acap-B*). A cDNA fragment of 17S rRNA was also amplified as an internal control using primers: 5′-CTTAAAGGAATTGACGGAA-3′ and 5′-ACGGGCGGTGTGTAC-3′. Genomic DNA was extracted from AX2 cells as described previously [49]. cDNAs corresponding to the full-length *arfA* and partial *acap-A* and *B* were isolated by SuperScript One-Step RT-PCR kit (Invitrogen, Carlsbad, CA). *ArfA* cDNA was amplified using primers: 5′-GCGCGCCATATGGGTCTCGCTTTTGGTAAACT-3′ and 5′-CGCGGATCCTTATTTTGAGGAGCTTGTTAAGGTAT-3′ and cloned into pET17b by NdeI/BamHI. A ∼3.2 kb fragment spanning from the 5′ end of *acap-A* cDNA was generated by RT-PCR then cloned into pCR4-TOPO vector by TOPO TA cloning kit (Invitrogen, Carlsbad, CA), and subsequently used as the template to amplify [400–845]ACAP-A (PZA) with primers: 5′-GCGCGCCATATGAATATTGAAAAGTATTTAGAAGAA-3′ and 5′-CGCGGATCCTTAATCAATTTCTTCACCTCTTAAGAT-3′. [400–845]ACAP-A was cloned into pET19b by NdeI/BamHI to obtain His_10_ [400–845]ACAP-A. [307–843]ACAP-B (PZA) was amplified from AX2 genomic DNA using primers: 5′-GCGGCTAGCCAAGATACATTATTTAAAAAAGGA-3′ and 5′-CGCGGATCCTTATTTGCAATTAATAACGAAACCTC-3′ and cloned into pET28b by NheI/BamHI to generate His_6_ [307–843]ACAP-B. Each construct was sequenced to ensure no undesired mutations introduced in the process of cloning.

### Protein Purification

*Dictyostelium* myristoylated and non-myristoylated ArfA were expressed in and purified from *Escherichia coli* BL21 (DE3) essentially following methods described for human Arf1 [25]. ArfA was co-expressed in *E. coli* BL21 (DE3) with N-myristoyltransferase in the presence of 10 µM myristic acid. The bacteria pellet from 2 L IPTG-induction culture was lysed in 20 mM Tris-HCl, pH 8.0, 100 mM NaCl, 1 mM MgCl_2_, 1 mM DTT and Complete™ protease inhibitor cocktail (Roche, Germany) using a French Press. The lysate was passed through a 5 ml HiTrapQ HP (GE Healthcare, Sweden). Myristoylated-ArfA (myr-ArfA) was purified from the Q-column flow through on a 25 ml phenyl-Sepharose HP column in a 50 ml NaCl gradient from 3000 to 100 mM. Fractions containing myr-ArfA were further purified by size exclusion using a Hiload 26/60 Superdex 75 column (GE Healthcare, Sweden) in 20 mM Tris-HCl, pH 8.0, 100 mM NaCl, 1 mM MgCl_2_, 1 mM DTT and 10% (v/v) glycerol. Myrisoylation of ArfA was verified by mass spectrometry with no detectable non-myristylated ArfA present in the sample (data not shown). His_10_ [400–845]ACAP-A and His_6_ [307–843]ACAP-B were expressed in *E. coli* BL21 (DE3) and purified from inclusion bodies using a method previously described [50] with some modifications. The bacteria pellet from 1 L IPTG-induction culture was resuspended in 35 ml 20 mM Tris-HCl, pH 8.0 with a complete protease inhibitor cocktail (Roche, Germany) and lysed by a French press operated at 12,000 psi. The lysate was clarified at 15,000×g for 10 min at 4°C. The pellet containing inclusion bodies was washed twice with 20 mM Tris-HCl, pH 8.0, 500 mM NaCl and 1 mM DTT and sonicated. Inclusion bodies were collected by centrifugation at 15,000×g for 10 min at 4°C then solubilized in 24 ml of 20 mM Tris-HCl, pH 8.0, 500 mM NaCl, 1 mM DTT, 5 mM imidazole and 6 M guanidine-HCl for 1 h at room temperature. Solubilized inclusion bodies were clarified at 15,000×g for 15 min at 4°C and the supernatant was applied to 1 ml HisTrap HP column (GE Healthcare, Sweden). Recombinant proteins were eluted by an imidazole gradient from 10 to 500 mM in 20 mM Tris-HCl, pH 8.0, 500 mM NaCl, 1 mM DTT and 6 M guanidine-HCl. The denatured His_10_ [400–845]ACAP-A or His_6_ [307–843]ACAP-B protein in the eluted fractions was refolded by step dialysis against urea concentration from 6 M to zero in 20 mM Tris-HCl, pH 8.0, 50 mM NaCl and 2 mM DTT. The precipitated protein was removed by centrifugation at 100,000×g for 15 min at 4°C and protein concentration of the supernatant was estimated by Bio-Rad assay (Bio-Rad Laboratories, Hercules, CA)

*Preparation of Large Unilamellar Vesicles (LUVs)*–All lipids were purchased from Avanti Polar Lipids (Alabaster, AL). LUVs consisted of 40% phosphatidylcholine, 25% phosphatidylethanolamine, 15% phosphatidylserine, 7.5% phosphatidylinositol, 10% cholesterol and 2.5% phosphatidylinositol 4,5-bisphosphate (PIP2) were prepared by extruding the lipid mixture through 1 µm pore polycarbonate membrane as described [51].

### GTP Binding

GTP binding to *Dictyostelium* ArfA was determined as described for human Arf1 with the no protein control subtracted as background [52]. Briefly, 1 µM of ArfA protein was incubated with 5 µM GTPγS and trace amount of [^35^S]GTPγS in 25 mM Tris-HCl, pH 7.5, 100 mM NaCl, 1 mM EDTA, 1 mM DTT and 0.5 mM LUVs at 30°C for various times. The reaction was stopped by diluting the samples into ice-cold 10 mM Tris-HCl, pH 7.5, 100 mM NaCl, 100 mM MgCl_2_ and 1 mM DTT. Protein-bound nucleotide was trapped on nitrocellulose filters and quantified by a scintillation counter.

### Arf GAP Assay

ArfGAP activity was determined by measuring GTP hydrolysis on Arf using an *in vitro* assay [53]. myr-ArfA protein was first loaded with [α-^32^P]GTP for 1 h at 30°C. Purified His-ACAP-A PZA or His-ACAP-B PZA was then incubated with ArfA preloaded with [α-^32^P]GTP in 25 mM HEPES, pH 7.5, 100 mM NaCl, 1 mM DTT, 2 mM MgCl_2_, 1 mM GTP and 0.5 mM LUVs at 30°C for various times in the time course experiments whereas in dose response experiments, PZA proteins were titrated into buffer and [α-^32^P]GTP-loaded ArfA for 1.5 h. The reaction was stopped as in “GTP binding” assay. Protein-bound nucleotide was released from the filters with formic acid, separated by thin layer chromatography on polyethyleneimine-cellulose plates and quantified using a Phosphor-Imager (GE Healthcare).

### Generation of Knock-Out Cells

Disruption of *acap-A* and *B* genes were performed using the Cre-*loxP* system as described [26]. A 5′ fragment of *acap-A* was amplified from AX2 genomic DNA by PCR using the forward primer 5′-AGTGGGCAACAACCAACAACAGAAATG-3′ and reverse primer 5′-GATGTTGAGGTTATATTATTGCCTGATGTT-3′. 3′ fragment was PCR amplified using primers: 5′-CATCG TAGTTTAGGTACTCATATCTCCAAG-3′ and 5′-TTCACCTCTTAAGATTTT CTCAGTTTCTTTTG. The PCR products were re-amplified with the addition of restriction enzyme sites and cloned into pLPBLP by SalI/SmaI (5′ fragment) and NdeI/BamHI (3′ fragment). This construct was linearized with SalI/BamHI, purified and electroporated into AX2 cells. Transformants were selected in HL-5 containing 10 µg/ml Blasticidin S. Independent clones were selected on bacterial lawns and screened for *acap-A* gene disruption by Southern hybridization. To create the *acap-A^−^/B* ^−^ double mutants, pDEX-NLS-cre was transiently expressed in the *acap-A* null cells and a Blasticidin and G418 double sensitive *acap-A* null clone was isolated to proceed with the disruption of *acap-B*. The 5′ and 3′ flanking fragments of *acap-B* were PCR amplified using the following primers: 5′-GTAATTTTAGAGAATTATTAGTAAGTATTA-3′; 5′-CTAGTTGCTTGAAGATAATCAGAGAAAACT-3′; 5′-CAACAAATAGAACATCACAAGATACATTAT-3′; 5′-GTTGATCCACATGAACTATTTAGTCCATTA. The PCR products were engineered with restriction enzyme sites and cloned into pLPBLP by SalI/HindIII (5′ fragment) and NdeI/BamHI (3′ fragment). This construct was linearized with SalI/BamHI, purified and electroporated into *acap-A* null cells double sensitive for Blasticidin and G418. Independent clones resistant to Blasticidin (10 µg/ml) were screened for *acap-B* gene disruption by Southern hybridization.

### Western Blotting

Lysates were resolved by 10% SDS-PAGE and transferred to nitrocellulose membranes. Membranes were blocked in 4% BSA in TBST (0.1% Tween 20 in Tris-buffered saline) overnight at 4°C, followed by incubation with the primary antibodies at 1∶2000 dilution as specified for 2 h at room temperature. Membranes were then washed and incubated with appropriate secondary antibodies for 1 h at room temperature. Proteins were visualized by enhanced chemiluminescence (GE Healthcare).

### F-Actin Staining and Confocal Microscopy

Cells were developed to the chemotaxis competent stage (5–6 h) and plated onto glass coverslips at ∼3500/mm^2^. Cells were allowed to adhere for 10 min and fixed with 1% formaldehyde, 0.125% glutaraldehyde, 0.01% TritonX-100 for 20 min. Subsequently, cells were permeablized with 0.1% saponin and 10% FBS then incubated with TRITC-phalloidin (Sigma-Aldrich, St. Louis, MO) for 25 min. Cells were washed 5 times with PBS and mounted in DakoCytomation Fluorescent Mounting Medium (Dako, Carpinteria, CA). Images were taken on a Zeiss LSM 510 attached to a Zeiss Axiovert 100 M with a 63×1.4 numerical-aperture plan Neofluar oil immersion lens (Carl Zeiss, Thornwood, NY) and processed with Adobe Illustrator software.

### Micropipette Chemotaxis Assay

The micropipette chemotaxis assay was performed as described previously [33]. Cells were developed to the chemotaxis competent stage (5–6 h) and plated onto Lab-TekII glass-bottomed chambers (Nalge Nunc International, Naperville, IL). The gradient was generated by a microinjector connected to a glass capillary needle filled with 1 µM cAMP (Femtotips, Eppendorf, Germany). The needle was brought into the field of cells and images were captured every 10 s with an inverted Zeiss Axiovert 200 microscope (Carl Zeiss, Thornwood, NY) equipped with automated filter wheels (Ludl Electronic Products, Hawthorne, NJ).

### Determination of Total Spore Yield and Viability

The amount of spores generated and the ability of the spores to germinate were measured according to a method described previously [38] with minor modifications. Briefly, cells were developed on agar for 48 h and the whole agar piece was transferred to 50 ml tubes containing 10 ml phosphate buffer (PB: 5 mM Na_2_HPO_4_ and 5 mM KH_2_PO_4_, pH 6.2). After vigorous vortexing, 1 ml of the suspension was transferred to 1.5 ml eppendorfs then treated with or without 0.5% SDS for 5 min and centrifuged at 1000×g for 5 min. The pellet was resuspended in 0.2 ml PB and spores were counted to determine the concentration. 50 µl of the 5000-fold diluted spore suspension and 30 spores were plated onto bacterial lawns to determine the total spore yield and viability respectively. The number of plaques were scored 3 days after plating and expressed as a percentage relative to plaques formed by wild-type spores. Each experiment was performed in triplicate.

### Spore Morphology

To visualize the spores in the spore heads, one fruiting body was isolated under a Leica stereoscope, flattened out with a piece of agar and imaged using a Zeiss LSM 510. For individual spore morphology, spores were harvested as described in “determination of total spore yield and viability” and resuspended in ∼30 µl PB. 3 µl of the spore suspension was spotted onto a rectangular coverslip and covered by a circle coverslip. Pictures were taken using an inverted Zeiss Axiovert 200 microscope (Carl Zeiss, Thornwood, NY).

## Supporting Information

Figure S1 ArfGAP homologs in *Dictyostelium*. Potential ArfGAPs in *Dictyostelium* were identified by either by using “ArfGAP” as keyword or by using human ASAP1 ArfGAP domain to search for homologous proteins in the *Dictyostelium* genome database. Next, protein domains of these 12 ArfGAPs were predicted using the Pfam database. *, the letters in bold above ArfGAP domains refer to the amino acid in place of the catalytic lysine. ‡, the cysteine residues in the ArfGAP domain of DDB0233656 are replaced by other amino acids as indicated on the graph.
(0.85 MB EPS)

Figure S2 Arf/Arl homologs in *Dictyostelium*. Alignment of human Arfs and Arf/Arl homologs in *Dictyostelium* using ClustalW. Arrow indicates the glycine residue that can be modified by myristoylation.
(1.46 MB EPS)

Figure S3 A schematic representation of the strategy to generate ACAP-A/B double knockout cells.
(0.89 MB EPS)

Video S1 Differentiated AX2 cells migrating towards a micropipette filled with 1 µM cAMP. Frames were taken every 20 s and presented at 15 frames/s (300x real time).
(4.06 MB AVI)

Video S2 Differentiated acap-A-/B (1–290) cells migrating towards a micropipette filled with 1 µM cAMP. Frames were taken every 20 s and presented at 15 frames/s (300x real time). Data shown are from 3 independent clones.
(3.48 MB AVI)

Video S3 Differentiated acap-A-/B (1–290) cells migrating towards a micropipette filled with 1 µM cAMP. Frames were taken every 20 s and presented at 15 frames/s (300x real time). Data shown are from 3 independent clones.
(4.91 MB AVI)

Video S4 Differentiated acap-A-/B (1–290) cells migrating towards a micropipette filled with 1 µM cAMP. Frames were taken every 20 s and presented at 15 frames/s (300x real time). Data shown are from 3 independent clones.
(4.25 MB AVI)
